# The application of new concepts of the assessment of the thyroid state to pregnant women

**DOI:** 10.3389/fendo.2022.987397

**Published:** 2022-08-16

**Authors:** Stephen P. Fitzgerald, Nigel G. Bean, Samuel P. Fitzgerald, Henrik Falhammar

**Affiliations:** ^1^ The Departments of General Medicine and Endocrinology, The Royal Adelaide Hospital, Adelaide, SA, Australia; ^2^ School of Medicine, The University of Adelaide, Adelaide, SA, Australia; ^3^ Adjunct Professor, School of Mathematical Science, University of Adelaide, Adelaide, SA, Australia; ^4^ Department of Obstetrics and Gynaecology, The Townsville Hospital, Townsville, QLD, Australia; ^5^ School of Medicine, University of Queensland, St Lucia, QLD, Australia; ^6^ Department of Endocrinology, Karolinska University Hospital, Stockholm, Sweden; ^7^ Department of Molecular Medicine and Surgery, Karolinska Institutet, Stockholm, Sweden

**Keywords:** pregnancy, thyroid physiology, FT4, TSH, thyroid state

## Abstract

Recently proposed concepts regarding the nature and assessment of the thyroid state have provided a model more consistent with empiric evidence. It now appears likely that there are no such entities as thyroid set points and individual euthyroidism. Rather than there being discrete thyroid states, peripheral organ parameters are associated with thyroid function in a continuous manner. Thyroid hormone levels and, in particular, levels of free thyroxine now appear to be superior to thyrotropin levels as indicators of the thyroid state. Complicating the assessment of the correlations of the thyroid state with pregnancy outcomes are the contribution of the placenta to maternal thyroid function, fetal thyroid development, the multiple potential pathways to any particular outcome, the likely presence of small critical periods of time, the differing genetics of fetal and maternal tissues, and the unreliability of thyroid hormone assays. Nevertheless, there is no apparent reason for there to be a change in pregnancy to the basic principles of thyroid hormone action. The relationships between mild abnormalities of the thyroid state and pregnancy outcomes and the value of treating such mild abnormalities remain uncertain and controversial. The evidence suggests that further investigation of these clinical questions might better be based on thyroid hormone, particularly free thyroxine, levels. In the investigation of borderline low thyroid states, the categories of subclinical hypothyroidism and isolated hypothyroxinemia might both be abandoned with attention being directed to low free thyroxine levels regardless of the thyroid-stimulating hormone (TSH) levels. For these changes to occur, there would ideally be improvements in the assays for free thyroxine in pregnancy. The evidence suggests that, just as in the non-pregnant situation, pregnancy guidelines based on thyrotropin levels may need revision.

## Introduction

Recent clinical evidence (1–3) has indicated that developments in the understanding of thyroid physiology (4–7) have implications for clinical thyroid medicine and thyroid research (8). In brief, the suggested conceptual changes are that clinical parameters associate in a continuous manner with thyroid function even throughout the normal range (3), that there are no such entities as thyroid hormone set points and “individual euthyroidism” (5), and that clinical parameters are more likely to be associated with thyroid hormone, particularly free thyroxine (FT4), levels than with thyrotropin (TSH) levels (1) such that the thyroid state of an individual is better judged biochemically by FT4 levels rather than TSH levels. It follows that subclinical thyroid dysfunction is not an accurate guide to the thyroid state of peripheral tissues and that trials of treatment of mild thyroid dysfunction might best be addressed at restoring FT4 levels rather than TSH levels (8).

Most of the research underpinning the above concept has focused on the non-pregnant state. Nevertheless, the meta-analysis of the associations of clinical parameters with the different thyroid function tests did include pregnancy outcomes, and the relevant results were identical to other clinical parameters in that FT4 levels, as compared with TSH levels, were more often associated with outcomes (1).

Though there is evidence to suggest that some of the associations between mild thyroid dysfunction and clinical outcomes are on the basis of causation, there is no convincing evidence that treatment of mild thyroid dysfunction is effective (9, 10). This is true for both pregnancy- and non-pregnancy-associated outcomes (11–13). Trials of treatments thus far may have been flawed however in that the vast majority have selected subjects on the basis of subclinical thyroid dysfunction and directed treatments to TSH levels rather than FT4 levels. Nevertheless, at least some of the associations may result from reverse causation or bidirectional causation (14) in which case the prospects of deriving benefit from treatment are reduced.

The approach to thyroid dysfunction in pregnancy may even be more controversial than in the non-pregnant state. For example, much thyroid/pregnancy research has been devoted to the associations of subclinical thyroid dysfunction with hypertensive disorders of pregnancy and (pre)eclampsia (15, 16), but a recent review of preeclampsia from a non-endocrinology center did not even mention thyroid dysfunction (17). On account of the relevant studies being negative or conflicting (12, 18, 19), national colleges of obstetrics do not consider subclinical thyroid dysfunction to be a relevant entity (20, 21). Nevertheless, increasing numbers of pregnant women are having thyroid treatments (22) presumably in part on account of guidelines (23–25) and some positive trials concerning both associations of subclinical hypothyroidism with pregnancy outcomes and treatment of the thyroid state (26–29).

Though our meta-analysis (1) did suggest that the new concepts and principles apply similarly to pregnant women, thyroid physiology of the pregnant state is more complex than in the non-pregnant state. Furthermore, a recent meta-analysis of thyroid function test associations with preeclampsia has suggested that there is a greater association with TSH levels than with FT4 levels (16). Here, we review and analyze the current state of the literature with regard to the extrapolation of the new general principles of assessment of the thyroid state to pregnant women.

## Physiology/pathophysiology

In the non-pregnant state, the outline of physiology is relatively simple in that the thyroid gland secretes thyroid hormones in response to TSH and these hormones feedback negatively on pituitary TSH secretion. Subsequently, clinical parameters may or may not be influenced by the levels of the thyroid hormones. The details of this system, e.g., receptor and deiodination physiology modulating local actions of thyroid hormones (30), add a layer of complexity as do the possibilities that TSH itself may influence some peripheral tissue physiological processes (31, 32) and that some peripheral tissues may modulate TSH secretion (introducing reverse or bidirectional causation) (14).

In pregnancy, the physiology is more complicated. The basic outline of maternal thyroid regulation is directly affected by the placenta *via* the secretion of human chorionic gonadotropin (hCG) (33) and probably too by other substances including antiangiogenic factors, particularly soluble fms-like tyrosine kinase-1 (sFlt1) (16). Although the thyrotrophic potency of hCG is low compared to TSH, high levels of hCG as seen in the first trimester result in significant thyroid stimulation (34). Later in pregnancy, hCG levels fall as do thyroid hormone levels (which drop below baseline) (35). The rises in total T4 (TT4) resulting from pregnancy-induced changes to hepatic stimulation of thyroid-binding globulin (TBG), albumin, and other proteins are more apparent than any rises in free thyroid hormones (35). The precise quantification of FT4 levels is however limited in pregnancy by assay performance (23, 36, 37). Multiple pregnancy, particularly in association with assisted reproductive technology, results in greater increases in hCG levels and thyroid stimulation as compared with singleton pregnancies (38). Furthermore, the placenta in abnormal conditions may secrete abnormal (15, 39), more potent forms of hCG in terms of thyroid stimulation.

The fact that thyroid hormone levels may be affected by placental function introduces the possibility of reverse causation, i.e., associations of any pregnancy parameters with the thyroid status may be by virtue of the thyroid hormone level and the parameter both being consequences of placental function (34). There is in fact empiric evidence (albeit conflicting) suggesting direct relationships between hCG levels and pregnancy outcomes (15, 40, 41). Potentially, there may be bidirectional influences, e.g., placental function may be influenced by the thyroid state and *vice versa*. Perhaps the initial influence is exerted by the preconception thyroid state (42).

The relationships between thyroid function and pregnancy outcomes may also be subject to confounding. For example, antibody positivity may affect thyroid function in pregnancy (43), but pregnancy outcomes may also be affected by antibody actions unrelated to any effect on thyroid function (23, 26).

In early pregnancy, there is increased iodine intake requirement on account of increased urinary iodine clearance (44) related to increased renal plasma blood flow and glomerular filtration (45). Throughout pregnancy, iodine is shifted from the maternal circulation to the fetal–placental unit (44). Iodine deficiency results in changes to thyroid function and maternal goiter or thyroid enlargement (46, 47). In turn, the changes to thyroid function in pregnancy are believed to be a cause of any related impairment of fetal neurological development (48). However, the evidence for this being the only mechanism at play is inconsistent (49), and it may be that deficits are more related to the iodine status than the thyroid status (50). Obesity, iron deficiency, and toxic chemicals may also result in changes to thyroid function in pregnancy and potentially might also have direct influences on pregnancy outcomes (37) . In these circumstances, the thyroid function may be a marker of the factors influencing pregnancy outcome, and correction of the thyroid state would not be expected to be therapeutic.

The assessment of the relationship between thyroid function and clinical parameters is also complicated by the fact that different pathophysiologies with different relationships to thyroid function may result in the same clinical parameter. This potentially can occur in non-pregnant and pregnant individuals. For example, high levels of thyroid hormones may directly increase the risk of atrial fibrillation (3), while low levels might increase the risk *via* an association with obesity (51). In pregnancy, preeclampsia, for example, might be related to low thyroid hormone-related endothelial cell dysfunction associated with decreased production of vasoactive substances (16, 52) or to high thyroid hormone-related endothelial cell dysfunction associated with impairment of mechanisms protective against endothelial damage (16, 53).

The opportunity for such multiple pathways increases in pregnancy given that the thyroid state can predispose to an outcome *via* direct effects on the fetus, *via* direct effects on the placenta, and/or indirectly *via* maternal effects (54). Possible examples include infant IQ that might be compromised directly by low thyroid hormone levels and by high thyroid hormone level-related placental dysfunction.

There may be critical periods during pregnancy where outcomes (37) and treatments (55) are sensitive to the thyroid status. Studies that miss these time points may potentially miss the relevant outcome (56). Studies in non-pregnant individuals are not so timing-dependent.

In pregnancy, apart from the complication of placental effects on maternal thyroid function, there is the complication of the presence of two thyroids, i.e., the fetal and maternal thyroids. It is quite possible that as well as there being placental effects on maternal thyroid function, there is control of fetal thyroid function by maternal thyrotrophin-releasing hormone (TRH) (54, 57). In these circumstances, low maternal thyroid function could conceivably lead to increased fetal thyroid function *via* higher TRH levels. Both the capacity of the fetal thyroid and the timing of its onset of function sometime in the second trimester (54, 58) potentially affect any outcome related to the maternal thyroid status. Prior to the maturation of fetal thyroid production, the FT4 that reaches the fetal brain is all of maternal origin (54, 58). The quantity of this FT4 is small, most FT4 (and FT3) having been deiodinated in the placenta (54, 59). This contrasts with the end of pregnancy when circulating fetal thyroid hormones are a mixture of hormones from the maternal and fetal thyroid glands (54).

The thyroid status of the placenta may not be a simple concept, with there being evidence that placental exposure to thyroid hormones may not be uniform with the maternal placenta being exposed to greater levels than the fetal placenta (59).

Thus, though maternal tissues, the maternal placenta, the fetal placenta, and the fetus are likely to have intracellular thyroid hormone levels related to the maternal circulating levels of thyroid hormones, this relationship is more complicated than in the circumstances of considering outcomes in non-pregnant individuals.

The complexity of the causation of the outcomes in pregnancy, and the multiplicity of tissue thyroid states to consider, may contribute to the conflicting results of associations of pregnancy outcomes with hormonal status while obscuring any potential intervention targets.

## Free thyroxine (FT4), thyroid-stimulating hormone (TSH), or total thyroxine (TT4) to assess the thyroid state in pregnancy

We have indicated that, in general, FT4 is the better indicator of the thyroid state of peripheral tissues (1). In our meta-analysis, FT4 performed better in all examined clinical parameters including death (1). Groothof et al. (2) confirmed this latter finding. There was evidence that at least some of the associations we observed were causal, these being the only associations that logically can be used to assess the thyroid state. We noted that even in the conservative circumstances of none of the associations being causal, there remained no evidence for the superiority of TSH. Our meta-analysis findings for pregnancy-related outcomes were similar to those for other parameters, but relatively few pregnancy studies were included (1).

At the most fundamental level, the physiology of thyroid hormone levels does not change with pregnancy. Thyroid hormone and TSH levels are determined by the thyroid hormone (principally FT4)/TSH feedback loop. The evidence indicates that the levels represent balance points of the physiological processes at play rather than the previous concept that the levels are genetically set and physiological processes act to defend these levels (5). The changes in pregnancy concern the limbs of the feedback loop, i.e., the details change. In particular, in pregnancy, the thyroid curve, the description of the FT4 response to TSH stimulation (7), changes in that on account of hCG, higher FT4 levels are seen for any level of TSH. The fundamental principle as to the derivation of the balance point for thyroid function tests and thereby the conclusion that FT4 is the better guide to the thyroid state is not affected by such changes.

It has also been previously thought that the genetically determined “set-point” of thyroid hormones of each individual represented an optimum level for the individual (7). It followed from this that on account of the sensitivity of TSH levels to primary changes in thyroid hormone levels, TSH levels were the best guide to any deviation from the individual set point and thereby the best guide to the thyroid state of peripheral tissues (60). The evidence now indicates that as well as the concept of such a set point being flawed (5), all levels of thyroid hormones within the normal range are associated with risks and benefits (61). Thus, though TSH levels may provide evidence of a change in thyroid function, there is no rationale to consider such a change to be necessarily disadvantageous, and though TSH levels may provide prognostic information, there is no reason to consider them to be the best guide to the thyroid state of peripheral state in general (8, 61). As a maternal TSH level thereby cannot be assumed to be the best biochemical marker of the thyroid state of even maternal tissues, it is difficult to sustain the argument that a maternal TSH level can be the best marker of the thyroid state of maternal and placental/fetal tissues given that they have different DNA and thereby potentially different sensitivities to thyroid hormones and different physiologies.

T4 is found in coelomic fluid in early pregnancy in proportion to maternal levels (58) and the placenta is impermeable to TSH (54). It follows that it is likely that the placenta and the fetus respond to an FT4 level regardless of the maternal TSH level. It is therefore likely that, notwithstanding the complexities of pregnancy physiology, levels of maternal FT4 are the better markers of the thyroid state of the mother and the placenta/fetus. As there exist physiological processes that act to minimize interindividual and intraindividual thyroid hormone level deviation from levels around the middle of the population range, and pathology tends to increase with such deviations, it is likely that optimum levels are likely to be mid-range (62, 63).

The recent meta-analysis by Toloza et al. (16) suggested a better relationship of a clinical parameter, preeclampsia, with TSH levels as compared with FT4 levels. Previous such studies of pregnant women, however, have shown similar but inverse relationships with FT4 and TSH (64, 65), and abnormally low TSH levels in the presence of normal FT4 levels (subclinical hyperthyroidism) are not associated with the pregnancy risks seen with elevated levels of thyroid hormones (overt hyperthyroidism) (66).

In pregnancy, therefore, we have conflicting studies both in terms of outcomes and in terms of the relative association with TSH or FT4 with these outcomes in the context of a lack of strong evidence as to whether the nature of any association is causal due to reverse causation or due to another mechanism.

On theoretical physiological grounds, TSH was previously thought to be the best indicator of the thyroid state. We have indicated above that now the physiological evidence would favor FT4.

On the other hand, there are problems with most if not all FT4 assays in pregnancy (23, 36, 37). FT4 represents only about 0.03% of circulating TT4. The challenges of measuring this small fraction increase in pregnancy on account of the increases in circulating levels of TBG, nonesterified fatty acids, and the decrease in circulating levels of albumin (23, 37). There is poor correlation between the readily available immunoassays, and even equilibrium dialysis and ultrafiltration followed by liquid chromatography and spectrometry have technical and practical limitations for use in pregnancy (37). The use of the FT4 index as a measure of FT4 may be superior, but this test is not widely available (37). There are recommendations to use trimester-specific, method-specific, and locally derived ranges. This latter suggestion may be impractical (23). These problems with the FT4 assay in pregnancy may blunt any associations with FT4 while sparing the associations with TSH. That is, any better relationship with TSH may be artifactual. In these circumstances, it may be pragmatic to prefer the TSH level even though strictly speaking any relationship might be stronger with the true FT4 level.

In addition, if indeed a relationship between thyroid function is a result of reverse or bidirectional causation, TSH may have a better association with an outcome than FT4 levels. An example of such might be if there were a causal relationship between low thyroid function and a parameter and, at the same time, a reverse relationship by which the parameter (perhaps as a compensation) stimulated thyroid function *via* the stimulation of TSH. In these circumstances, on account of the initial causation, the parameter would initially be associated with low FT4 levels and high TSH levels, the association with the high TSH levels being on account of the inverse correlation in the population between FT4 levels and TSH levels (67). The reverse causation relationship however would subsequently raise the TSH levels and thereby the FT4 levels. In this situation, the initially high TSH levels have been pushed higher but the initially low FT4 levels are not so low and thus the relationship has become stronger with TSH levels. The associations of obesity with TSH and FT4 levels may be an example of this type of bidirectional causation (14). The study by Toloza et al. (16) is potentially a similar example as again the relationship demonstrated, between thyroid function and gestational hypertension/preeclampsia, concerned a relationship with low thyroid function.

Any relationship of an outcome with TSH might also potentially result from a relationship between TSH and any other parameter, e.g., hCG (34, 40, 41) or thyroid antibodies (23, 43, 68), that affects outcomes. If such a relationship was stronger than the corresponding relationship with FT4, the relationship between TSH levels and outcomes might be stronger than the relationship between FT4 and outcomes. In these circumstances, the better relationship with TSH would be an example of confounding.

Finally, a stronger association with TSH might be seen should TSH directly act on placental or fetal tissues. In the non-pregnant state, this phenomenon has been recognized. TSH has been reported to have effects on the heart (31) and on bone (32) independently of any effects mediated by TSH as an indicator of the thyroid state.

Despite the problems with FT4 assays and the possible causes of spurious TSH relationships, there are studies that report superior relationships with FT4 (68–71). There are fewer opportunities for such superiority of FT4 to be spurious.

It has been suggested that the use of TT4 with mathematical adjustment of the range to take into account the increase in TBG may be superior or an alternative to the use of FT4 in pregnancy (23, 37). For most of pregnancy, TT4 levels are 50% above baseline (23, 37). There are however disagreements regarding the detail of any such mathematical adjustments (72). There are also theoretical problems with this suggestion. In the first instance, a major factor determining the changes in the thyroid hormone-binding globulin, and thereby the TT4 level in pregnancy, is the placenta *via* the production of estrogen (73). Thus, the previously mentioned possibility of reverse causation of pregnancy parameters related to the placental production of hCG is amplified. That is, pregnancy outcomes and TT4 levels may result from placental function rather than the pregnancy outcomes being a consequence of the TT4 levels. In an individual case, the FT4 proportion of TT4 would be uncertain. The use of TT4 rather than FT4 contradicts the “free hormone hypothesis” that is thought likely to apply to thyroid hormones (as well as to sex steroids, glucocorticoids, and vitamin D) (74, 75). Furthermore, the modern studies looking at correlations of clinical parameters, including those related to pregnancy, with the thyroid status have relied on FT4 levels (37), and it is not known whether or not such correlations can be extrapolated to TT4 levels.

We have previously indicated that although clinical parameters appear to associate most robustly with FT4 levels, free and total T3 levels also associate with these parameters more often than do TSH levels and that further studies to determine the idiosyncrasies of each organ/tissue are indicated (61). Analogously, in the perhaps unlikely circumstances that TT4 levels were found to be the best guide to the pregnancy thyroid state, the fundamental principle would remain that thyroid hormone measurement is preferable to TSH measurement.

## Synthesis and conclusions

Ideally, studies of associations of clinical parameters with thyroid function tests would indicate the best test of the thyroid state relevant to the parameter, and ideally, the relationship between the thyroid state and the parameter would be causal and potentially amenable to intervention. Furthermore, it would be simpler if the relationship between the thyroid function and the parameter was in one direction only. One would then hope that follow-up intervention studies would have positive results, confirming the causal nature of the associations and the value of therapy.

With regard to the non-pregnant state, there is now a model of physiology coherent with empirical data (61). There is good evidence that FT4 is generally superior to TSH in defining the thyroid state, and there would appear to be good prospects for proceeding with new intervention trials using FT4 levels for subject selection and treatment targets. In particular, the associations in older individuals OF atrial fibrillation, dementia, and death WITH FT4 levels, apparently satisfying the above conditions, appear ripe for intervention studies. Notably, these associations concern FT4 levels at the upper end of the range. The nature of the associations at the lower ranges of thyroid function, where the impetus to treat in pregnancy lies, seems less clear.

The field of the association of the thyroid state with pregnancy-related outcomes appears to be less advanced and more conflicted. Consequently, many of the recommendations in the guidelines are weak or based on a modest evidence base (23–25). This state may be a result of the greater complexity of thyroid function associations with outcomes in pregnancy. Any state of thyroid abnormality may be a heterogeneous group of diseases (37), only some of which might benefit from thyroid function manipulation alone. It seems that we need more studies of the kind published by Korevaar et al. (15) in which a more wholistic view of thyroid physiology was employed, the analysis encompassing clinical parameters, indices of thyroid function and hCG. It may be too that such studies also need to consider other factors such as preconception indices of thyroid function (42), iodine status, and antibody status.

Our general conclusions regarding the implications of the recently introduced concepts of thyroid action and measurement included recommendations that the thyroid state be assessed on the basis of FT4 levels rather than TSH levels and that subclinical hypothyroidism not be regarded as an accurate measure of the thyroid state of peripheral tissues (8, 61). We have indicated that borderline low thyroid function is reflected in a borderline low FT4 level regardless of the TSH level. Isolated hypothyroxinemia, which may be a mild form of secondary hypothyroidism (76), then becomes a subset of borderline low thyroid function that also includes a subset of subclinical hypothyroidism. These conclusions conflict with the various current guidelines regarding diagnosis, monitoring, and treatment in pregnancy that are based on TSH levels (23–25).

There are no apparent reasons for the fundamental general principles of thyroid physiology not to hold in pregnancy, and the patchy empirical evidence does not indicate the state of pregnancy to be exceptional in this regard. It follows that, analogously to those pertaining to the non-pregnant state, all of the current guidelines, which differ in detail but are based on identical principles, may be flawed. Any true relationships between either or both TSH and subclinical thyroid dysfunction and clinical outcomes are likely to be indirect, reflecting the population correlation of TSH with FT4 (67). Otherwise, relationships may result from the opportunities for artifact, confounding, reverse causation, and bidirectional effect. If mild thyroid dysfunction truly affects pregnancy and fetal outcomes, diagnosis and treatment on the basis of FT4 levels are likely to be more effective than relying on TSH levels.

The largely negative trials to date performed in non-pregnant individuals on the basis of TSH levels confirm the impression that such subject selection criteria and treatment targets are less than optimal (9, 10). The few positive studies in pregnancy largely involve antibody-positive women and are difficult to interpret mechanistically (23). It is difficult to separate any effects of treating the low thyroid state *per se* from any non-specific/pharmacological effects of thyroxine supplementation particularly in antibody-positive women. Though it has been proposed that the negative effect of thyroid antibodies on hCG stimulation of thyroid function provides a potential mechanism for the link between thyroid antibodies and pregnancy outcomes (43), this logic would imply similar outcomes in individuals with similar thyroid hormone levels in the absence of antibodies. New trials of different designs appear indicated. These may depend on the more sophisticated understanding of the associations between thyroid function and pregnancy outcomes suggested above.

It may be, that though apparently safe (77), intervening in cases of borderline thyroid dysfunction in pregnancy, however defined, offers modest or even no benefit (11, 12, 20, 21). In these circumstances, the definition of borderline thyroid dysfunction in pregnancy would have limited clinical importance. If this could be clearly demonstrated, clinicians and researchers could direct their attention elsewhere.

On the other hand, there are studies of isolated hypothyroxinemia that do suggest that borderline low thyroid function may have significant associations with placental abruption (13), spontaneous premature delivery (68), and perhaps most likely with disorders of fetal neurodevelopment (37, 78). Inconsistent definition of borderline thyroid dysfunction and the timing of testing and intervention may have contributed to this conflict in results (37, 56). Furthermore, animal data suggest that the association of hypothyroxinemia and abnormal fetal neurodevelopment is causal (54) and treatable (55). These conclusions are not so strong for humans (11, 54).

Regardless of the relevance of borderline low thyroid function to pregnancy outcomes, there is at this stage no apparent physiological reason or empiric evidence justifying the continued TSH level approach to classifying and managing the thyroid state in pregnancy. If anything, TSH levels would appear to be less accurate in pregnancy than in the non-pregnant state. Thyroid hormone levels and, in particular, FT4 levels would appear to be more accurate.

Although there are already studies supporting the superiority of FT4 levels in the diagnosis of the thyroid state in pregnancy (68–71), a more accurate assay for FT4 in pregnancy would likely aid a shift in emphasis from TSH levels to FT4 levels. In the absence of such an assay, it may be necessary in some circumstances to continue to use TSH levels for pragmatic reasons and advancement in thyroid/pregnancy research may be limited. Perhaps, previously, the need for an accurate measurement of FT4 in pregnancy was not thought to be so pressing given that TSH was thought to be the best guide to the thyroid state. One recommendation following the changing concepts regarding the thyroid state might therefore be to further research FT4 assays in pregnancy. Failing this, the value of TT4 measurement might be explored further.

In conclusion, our review found that despite much work, there is not clarity in the current status of the interaction of thyroid function (and in particular borderline low thyroid function) with pregnancy outcomes. The physiological models and empiric evidence are not mutually coherent. These deficiencies may originate in part from the fundamental emphasis on TSH levels in the assessment of the peripheral thyroid state. We found no reasons to consider that in pregnancy, the conceptual changes regarding the nature of, and the general assessment of, the thyroid state do not apply. By extension, the clinical and research implications for pregnancy might parallel the general implications (8) and include deemphasizing arbitrary categories, and in particular the concepts of subclinical hypothyroidism and isolated hypothyroxinemia, with a switch of emphasis to FT4 levels as a continuum, regardless of TSH levels.

In pregnancy, the complex physiology/pathophysiology and the difficulties of obtaining an accurate measurement of FT4 complicate the application of these implications.

## Author contributions

STF Conceived paper, wrote initial draft. NB provided mathematical support, revised the draft. SAF collaborated with review of obstetric physiology/fetal development. HF revised draft substantially. All authors contributed to the article and approved the submitted version.

## Conflict of interest

The authors declare that the research was conducted in the absence of any commercial or financial relationships that could be construed as a potential conflict of interest.

## Publisher’s note

All claims expressed in this article are solely those of the authors and do not necessarily represent those of their affiliated organizations, or those of the publisher, the editors and the reviewers. Any product that may be evaluated in this article, or claim that may be made by its manufacturer, is not guaranteed or endorsed by the publisher.
